# Plasticity of the Systemic Inflammatory Response to Acute Infection during Critical Illness: Development of the Riboleukogram

**DOI:** 10.1371/journal.pone.0001564

**Published:** 2008-02-13

**Authors:** Jonathan E. McDunn, Kareem D. Husain, Ashoka D. Polpitiya, Anton Burykin, Jianhua Ruan, Qing Li, William Schierding, Nan Lin, David Dixon, Weixiong Zhang, Craig M. Coopersmith, W. Michael Dunne, Marco Colonna, Bijoy K. Ghosh, J. Perren Cobb

**Affiliations:** 1 Center for Critical Illness and Health Engineering, Department of Surgery, Washington University in St. Louis, St. Louis, Missouri, United States of America; 2 Department of Anesthesiology, Washington University in St. Louis, St. Louis, Missouri, United States of America; 3 Department of Computer Science and Engineering, Washington University in St. Louis, St. Louis, Missouri, United States of America; 4 Department of Mathematics, Washington University in St. Louis, St. Louis, Missouri, United States of America; 5 Department of Genetics, Washington University in St. Louis, St. Louis, Missouri, United States of America; 6 Department of Molecular Microbiology, Washington University in St. Louis, St. Louis, Missouri, United States of America; 7 Department of Pathology and Immunology, Washington University in St. Louis, St. Louis, Missouri, United States of America; 8 Department of Electrical and Systems Engineering, Washington University in St. Louis, St. Louis, Missouri, United States of America; University College London, United Kingdom

## Abstract

**Background:**

Diagnosis of acute infection in the critically ill remains a challenge. We hypothesized that circulating leukocyte transcriptional profiles can be used to monitor the host response to and recovery from infection complicating critical illness.

**Methodology/Principal Findings:**

A translational research approach was employed. Fifteen mice underwent intratracheal injections of live *P. aeruginosa*, *P. aeruginosa* endotoxin, live *S. pneumoniae,* or normal saline. At 24 hours after injury, GeneChip microarray analysis of circulating buffy coat RNA identified 219 genes that distinguished between the pulmonary insults and differences in 7-day mortality. Similarly, buffy coat microarray expression profiles were generated from 27 mechanically ventilated patients every two days for up to three weeks. Significant heterogeneity of VAP microarray profiles was observed secondary to patient ethnicity, age, and gender, yet 85 genes were identified with consistent changes in abundance during the seven days bracketing the diagnosis of VAP. Principal components analysis of these 85 genes appeared to differentiate between the responses of subjects who did *versus* those who did not develop VAP, as defined by a general trajectory (riboleukogram) for the onset and resolution of VAP. As patients recovered from critical illness complicated by acute infection, the riboleukograms converged, consistent with an immune attractor.

**Conclusions/Significance:**

Here we present the culmination of a mouse pneumonia study, demonstrating for the first time that disease trajectories derived from microarray expression profiles can be used to quantitatively track the clinical course of acute disease and identify a state of immune recovery. These data suggest that the onset of an infection-specific transcriptional program may precede the clinical diagnosis of pneumonia in patients. Moreover, riboleukograms may help explain variance in the host response due to differences in ethnic background, gender, and pathogen. Prospective clinical trials are indicated to validate our results and test the clinical utility of riboleukograms.

## Introduction

Critical illness is marked by organ dysfunction, the need for vital support, and a high risk of death, occurring against a backdrop of systemic immune activation. This immune activation may begin as an adaptive response to the initial injury, however, as the disease progresses, the immune response may become maladaptive or paralyzed [1], [2]. Critical illness-associated immune dysregulation has been described as the interplay between pro- and anti-inflammatory responses [3], although recent evidence suggests a mixed inflammatory state is common [4], [5]. While this process has been qualitatively described, there are no quantitative diagnostic or prognostic tools that have been validated clinically to assess immune status in the critically ill [6]. Consequently, infectious complications are not only common in intensive care units but also difficult to diagnose [7]. This has contributed to inappropriate use of broad-spectrum antibiotics and the emergence of multi-drug resistant organisms [8], [9].

A few years ago, studies employing cultured human cells suggested that instead of a single molecule (*e.g.*, IL-6), a constellation of molecules could be used to monitor the complexities of the inflammatory response, serving as markers of infection [10], [11]. Miniaturized, multiplexed assays provide a rapid method for the unbiased screening of thousands of molecular species in a single assay [12]. These technological advances provided the potential for investigators to leverage high-throughput assays to better study the host response to and recovery from critical illness and injury [13], [14]. Improved molecular diagnostics and prognostics, a better understanding of the complexity of systemic inflammation, and new therapeutic targets are expected deliverables, as reviewed recently [4], [12].

Based upon our ability to diagnose abdominal sepsis in pilot mouse studies [15], we hypothesized that the host response to infection could not only differentiate between infected and non-infected states, but could also be used clinically to differentiate between the host response to infectious agents and to model the host response to and recovery from infectious perturbations. Pneumonia was chosen as an immune system perturbation, given its relative frequency and considerable cost in terms of patient morbidity and health care expense [7], [16]. A bench-to-bedside, translational approach was employed to study the host response to pneumonia in critically ill subjects, comparing the informational content of standard clinical parameters and plasma cytokines to changes in the RNA abundance in circulating leukocytes.

## Methods

### Mice, experimental procedures, and samples

Care and use of laboratory animals were conducted in accordance with a protocol approved by the Washington University Animal Studies Committee, in compliance with guidelines (N01-RR-2-2135) prepared by the Committee on Care and Use of Laboratory Animals, Division of Research Resources, National Institutes of Health. Seven to nine week-old, male C57BL/6 mice were purchased (Harlan, Inc. Madison WI) and allowed to acclimatize for at least one week in a temperature- and light-controlled, pathogen-free barrier facility. Treated animals and concurrently studied controls were observed at 24 hour intervals for survival over eight days. In additional cohorts, whole blood was collected at 24 hours after injury.

The 5 experimental groups were selected to reflect clinically important distinctions relevant to care of ICU patients: high mortality Gram-negative pneumonia with *Pseudomonas aeruginosa* (40 µl of 0.3 McFarland culture, 90% 7-day mortality (∼2–4×10^7^ organisms)), high mortality Gram-positive pneumonia with *Streptococcus pneumoniae* (60 µl of 0.5 McFarland culture, 85% 7-day mortality (∼1.8–3.6×10^7^ organisms)), and moderate mortality Gram-negative pneumonia with *Pseudomonas aeruginosa* (20 µl of 0.1 McFarland culture, 50% 7-day mortality (∼2–4×10^6^ organisms)). To induce severe systemic inflammation without infection, intratracheal (i.t.) injection of *P. aeruginosa* lipopolysaccharide (500 µg in 50 µl 0.9% normal saline; Sigma, St. Louis) was performed (LPS group, 90% 7-day mortality). Mice injected i.t. with normal saline vehicle acted as the concurrent control group (saline group, 0% 7-day mortality). Previously reported protocols were used to intratracheally instill fluid into the lung [17], [18]. The census of surviving mice was recorded at 24-hour intervals for seven days.

In three additional cohorts of animals, blood was collected into an EDTA-coated syringe from the inferior vena cava being careful to avoid contamination of the needle with other tissues. Blood was diluted 1∶1 with normal saline, pooled for the 8 animals in each treatment group, and separated into cells and plasma. Plasma was stored at −80°C until use. Erythrocytes were lysed hypotonically and RNA from peripheral leukocytes was harvested using RLT (Qiagen) and stored at −80°C until use. The 24 hour time point after injury was chosen as a time before appreciable mortality develops in animals with significant lung injury.

### Target cRNA and gene expression signal

Each RNA sample was run on one GeneChip (a total of 15 mouse blood GeneChips from 120 animals). Total RNA from mouse blood was extracted as previously described [19]. cRNA target for GeneChip hybridization was prepared from total RNA (Affymetrix, Santa Clara, CA). Both total RNA and cRNA were electrophoretically assessed for quality (Agilent Bioanalyzer). The mouse blood cRNA samples were hybridized with the U74Av2 GeneChip (approximately 12,400 probe sets). Fluorescent hybridization signal was detected using a GeneChip Scanner 3000 (Affymetrix). These mouse microarray data (and those for patients, see below) have been deposited in NCBI's Gene Expression Omnibus (GEO, http://www.ncbi.nlm.nih.gov/geo/) and are accessible through GEO Series accession number GSE6377.

### Data analysis and statistical tests for differential expression

Expression values were calculated from GeneChip .cel files using Robust Multichip Average (RMA) software [20]. Differentially expressed genes were identified using a mixed-model analysis of variance (ANOVA) and linear contrasts (Partek® Infer™ software) as previously reported [15]. Leave-one out cross-validation (k-nearest neighbors, k = 2) was used to determine the reproducibility within this experimental set. Principal components analysis (PCA) was used to visually explore global effects for genome-wide trends, unexpected effects, and outliers in the expression data (Partek® Pro™ software, www.partek.com).

#### Patient studies

After obtaining informed consent, venous blood (7 ml) was collected from mechanically ventilated, non-septic patients according to a protocol approved by the Washington University Institutional Review Board (#2004-0294). Patients were candidates for enrollment if they were on mechanical ventilation in the surgical ICU medical ICU, neurological ICU, or cardiothoracic ICU (CTICU) for 48 hours, were expected by the attending ICU physician to need mechanical ventilation for at least another 48 hours at the time of enrollment, and could provide written informed consent (from the patient or legal surrogate). VAP was diagnosed by the ICU attending physician, consistent with recently reported recommendations [16], without input from the investigators. Clinical data were entered into a VAP database, including gender, ethnic background, age, admitting diagnosis, type of ICU, APACHE II score, airway sampling technique and culture results, initial antibiotic therapy, and maximal clinical pulmonary infection score (CPIS) calculated based upon available data (several patients lacked daily arterial blood gas measurements to calculate P_a_O_2_/F_i_O_2_ ratios) [21]. Patient blood was processed as described previously to minimize red blood cell RNA artifact [13]; briefly, samples were centrifuged (400×g 10′ RT) to form a buffy coat and to separate plasma from cells. Plasma was withdrawn and stored at −80°C until use. Blood cells were diluted into EL buffer (90 ml) (Qiagen) and incubated on ice for 15′. Leukocytes were pelleted by centrifugation (400×g 10′ 4°C), washed with EL buffer (30 ml) and lysed into RLT buffer (Qiagen) containing 1% β-mercaptoethanol. Genomic DNA was sheared by repeated passage through an 18 gauge needle and the resultant material was stored at −80°C.

#### Patient plasma cytokine analysis

Cytokines (GRO-α, IFN-γ; IL1-β; IL1Ra; IL1sr2; IL4; IL6; IL8; IL10; IL12; IL18; MCP1; MIP1α; MIP1β; NGF; RANTES; TNFα; TNF-sr1; TNF-sr2) were measured using a microarray immunoassay as previously described [22]. Procalcitonin was measured according to the manufacturer's instruction (BRAHMS PCT LIA kit, Product number 354.1).

#### Patient blood leukocyte mRNA profiling

RNA was extracted, amplified and assessed for quality as described for murine samples. cRNA was hybridized against the HG-FOCUS array (Affymetrix, >8700 probe sets encoding ∼8400 genes) and imaged as described for murine samples. Orthologs of murine genes were identified by comparison of the GeneChip Identifiers using the NetAffx Toolkit (Affymetrix). Consistent with recently published consensus statements [16], [21], clinical data were judged to determine when (if ever) each patient developed ventilator-associated pneumonia, with each patient acting as her/his own control. The timeline for each patient was defined such that the day of VAP diagnosis by the ICU attending physician was defined as day 0. A seven day time window from the gene expression time series was chosen as days −3, −1, 0, 1, and 3, with day 0 being the day that a patient was diagnosed as having VAP by the attending physician. Because blood samples were collected every other day, patients' samples were collected either on days −2, 0, +2 or on days −3, −1, +1, +3 relative to the VAP day of diagnosis described as time 0. For the purpose of analyzing the data from those patients who had samples collected on “odd” days, the time 0 data for these patients were interpolated. Those mRNA species whose abundance changed concordantly among the patients during the 7-day window surrounding the date of diagnosis were identified using extraction of Differential Gene Expression (EDGE) software [23]. Online databases were used to determine gene annotation and functional categorization (DAVID 2.0 accessed 16 November 2006) [24].

#### Clustering algorithm

Patient genes identified by EDGE were clustered as described previously [25]. Briefly, a gene co-expression network was constructed by connecting every gene to the top *d* genes (*d* = 5 in this study) to which its expression profile is most similar. The network then was partitioned into a set of communities, *i.e.*, relatively densely connected sub-graphs, by a spectral graph algorithm [26]. The genes within each community formed a cluster. The number of clusters was determined automatically by the algorithm in order to maximize a modularity score [25], [26]. Gene expression data were normalized prior to clustering such that the expression levels of each gene for each patient had a mean of zero and a standard deviation of one. Similarity between two gene expression profiles was measured by Pearson correlation coefficient.

#### Karhunen-Loeve decomposition of microarray gene expression data

To determine the dynamics of the host response to pneumonia, we constructed first a raw gene expression matrix corresponding to the *i*th pneumonia patient after RMA normalization [20] to be *X_i_* = [*x_i_*(1) … *x_i_*(*N_T_*)] where *x_i_*(*k*)∈ℜ*^N^*, (*k* = 1,…,*N_T_*, *i* = 1,…11, and *N* = 8793 genes) is the gene expression vector at *k* th time point. *k* = 1,…,*N_T_* where *N_T_* = 11 for most patients, correspond to sample collecting days 1, 3, 5, …, 21. Note that for few patients, only a portion of the time series (i.e., less than 11 points) was available.

For the EDGE-selected genes, the data were projected onto a smaller dimensional space using the series expansion method similar to principal components analysis, Karhunen-Loeve Decomposition (KLD) [27]. In order to preserve the alignment of the time series with respect to day 0 of VAP, we first obtained the average expression vectors 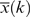, *k* = 1,2,…9, by averaging the expression values at days corresponding to −3,−1,…,13 in all patients (corresponding to the nine time points that most patients had samples collected). The KLD method looks for a basis *ψ*_1_,*ψ*_2_,…*ψ_N_* so that one can expand 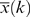 as
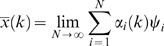
where 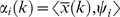 and 〈·,·〉 stands for the standard inner product.

The orthonormal basis *ψ*_1_,*ψ*_2_,…*ψ_N_* can be selected as the eigenvectors of the correlation matrix *C*_1_∈ℜ*^N×N^*, obtained as
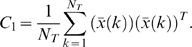
The first principal mode *ψ*_1_ corresponds to a constant bias term. Hence the most important variation is captured by *ψ*_2_,*ψ*_3_,…*ψ_N_* respectively in the decreasing order. Once the orthonormal basis is obtained, each patient data can be projected onto this basis as 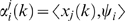 for patients *j = *1, 2, … . The discrete derivative of the coefficients 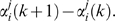



#### Validation of microarray results

Select genes were subjected to real-time quantitative PCR (RTq-PCR) for independent confirmation of relative expression levels. cDNA was generated and 100 ng was subjected to routine SybrGreen RT-PCR as per manufacturer's instruction (Applied Biosystems, Foster City, CA). In addition, 85 genes were selected at random from the total number on the GeneChip. The gene expression signal from these genes was analyzed in a manner identical to that described above. This procedure was repeated 100 times to estimate the informational value of randomly selected genes.

### Validation of the riboleukogram

After enrollment of the first 20 patients, a second cohort of 7 patients was analyzed to validate the informational content of the leukocyte RNA species (genes) that changed in abundance in response to critical illness complicated by VAP. The blood handling, processing, and GeneChip analysis protocols were identical to those described above.

## Results

### Murine model

**Figure 1:**
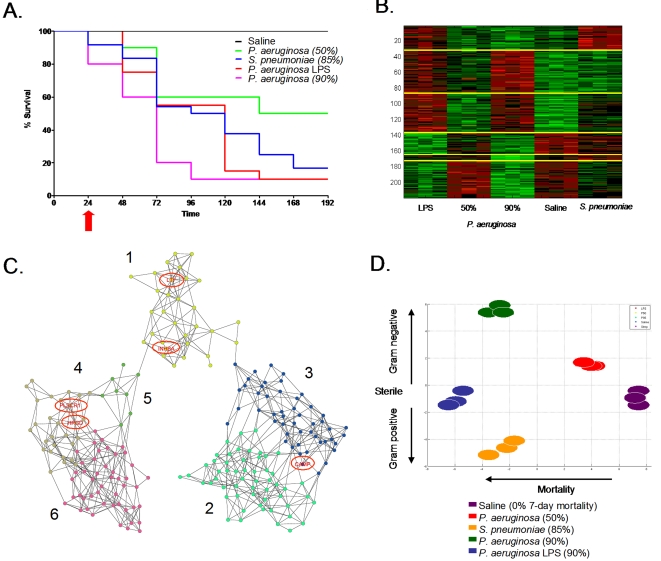
(A) Eight-day survival curves of mice challenged with intra-tracheal injection of one of 5 solutions, each dosed to produce the observed mortality. Significant differences (p<0.05) were observed between the 80–90% mortality (Pseudomonas bacteria, Streptococcus bacteria, and Pseudomonas LPS) *versus* 50% mortality (Pseudomonas bacteria) *versus* 0% mortality groups. Blood samples were obtained at 24 hours (red arrow), a time prior to appreciable mortality. (B) Clustering of the 219 probe sets that differentiated the five treatment groups separated the probe sets into six clusters. (C) Co-expression network analysis of these six mouse gene clusters were used to explore the gene expression cartography of the leukocyte response to pneumonia. Genes in common with the human coexpression network (Figure 3B) are circled. (D) Principal components (PC) analysis of an algorithm-selected subset of the 219 probe sets whose microarray-measured RNA abundance in leukocytes isolated 24 h after the onset of pneumonia. PC2 appeared to explain in part expression signal variance due to mortality rates, while PC3 explained in part the variance due to type of insult.

Intratracheal (i.t.) installation of saline caused no deaths over 7-days whereas i.t. introduction of *P. aeruginosa* endotoxin resulted in death of 90% of the animals studied within 96 hours (Figure 1A). The 7-day mortality caused by live *P. aeruginosa* was adjusted by varying the size of the inoculum and injuries causing 50% and 90% 7-day mortality were achieved. A dose of *S. pneumoniae* was given i.t. that resulted in 85% 7-day mortality. Once these injuries were established, three separate cohorts of mice were used for each experimental group in the subsequent studies.

Peripheral blood was collected and pooled from groups of 4–7 mice 24 h after surgery, prior to appreciable mortality in any group (Figure 1A). All murine RNA were of good quality based on the peak profiles of 18S and 28S ribosomal RNA. cRNA generated from these samples had a uniform size distribution. All hybridizations were of good quality; both the number of features present (35–40%) and the signals on each array fell within acceptable ranges [28].

Analysis of normalized gene expression data identified 219 probe sets (40 unannotated ESTs, 10 redundant probe sets, 169 annotated genes (Table S1) whose expression levels differentiated between the five groups. Leave one out cross-validation using k-nearest neighbors (k = 2) resulted in a 93% classification accuracy. The single misclassified sample was from the low-dose *P. aeruginosa* infection and was classified as “saline”. These 219 genes differentiated between the host responses to Gram-negative bacteria, Gram-negative bacterial toxin (LPS) and Gram-positive bacteria. The probe sets clustered into six groups (Figure 1B) and these groups defined the gene expression cartography of the murine response to pneumonia (Figure 1C). Genes that fell within clusters 2 and 3 exhibited increased RNA abundance in animals responding to high lethality insults. Genes that fell within clusters 4–6 were transcriptionally suppressed during high lethality insults. Cluster 1 bridged the two groups. Gene ontology assignments identified enriched molecular functions in the three distinct groups. The bridging cluster (cluster 1) was enriched for genes involved in the immune response (N = 10, P = 4×10^−7^) and genes with NTPase activity (N = 6, P = 5×10^−4^). Clusters 2 and 3 were enriched for genes involved in intracellular signaling pathways (N = 18, P = 6×10^−7^). Clusters 4–6 were enriched for genes that encode nuclear proteins (N = 24, P = 9×10^−4^). Principal components analysis of the microarray-derived transcript abundances of the genes selected based on cross-validation clearly differentiated the five experimental groups (Figure 1D) based on the 7-day mortality (principal component 1) and the type of agent used (Gram positive, sterile, Gram negative, principal component 2).

### Clinical study—training cohort

After being mechanically ventilated for >48 h, 27 patients were enrolled into the study. The first 20 patients enrolled were assigned arbitrarily to a training cohort; the other 7 were assigned to a validation cohort. Blood samples were taken at ∼48-hour intervals during the study period and then separated into plasma and leukocytes (see below). Of the 20 patients in the training cohort, eight patients either were extubated without developing VAP or withdrew from the study. Of the 12 patients in this cohort who developed microbiologically-confirmed VAP (Table 1), 11 met our analysis criteria of having samples before and after the attending physician made a diagnosis of VAP (that is, one patient, #9, was excluded from analysis for developing VAP on the study entry day). Clinical pulmonary infection scores (CPIS) increased in all 11 patients coincident with the diagnosis of VAP. Three of the patients were culture positive for a Gram positive agent (*S. aureus*) and the remaining eight patients were culture positive for one or more Gram negative agents. In every case, initial intravenous antibiotic therapy was appropriate for the cultured organism responsible for VAP, based upon cultured organism susceptibilities. Nine of the 11 patients developed VAP 3–6 days after enrollment in the study (“early VAP”) while two patients developed VAP after prolonged mechanical ventilation (“late VAP”, Table 1). All of the patients survived and were discharged from the ICU. Patient-specific timelines were aligned for analysis by assigning “day 0” to the day that the attending physician diagnosed VAP.

**Table 1:** Patient Characteristics.

Patient No.	Gender	Ethnicity	Age	Admit Diagnosis	ICU	APACHE II Score	VAP day	Maximum CPIS score	Culture Results	Initial Antibiotic Therapy
Training Set										
1	M	C	65	COPD	SICU	21	6	8*	*Serratia marcescens*	Cefepime, Fluconazole, Metronidazole, Vancomycin
2	M	C	21	MVC	SICU	24	6	6*	*Acinetobacter calcoaceticus-baumanii* and *Serratia marcescens*	Cefazolin, Cefepime, Clindamycin
3	M	C	69	CHI	SICU	21	4	6*	*Enterobacter aerogenes*	Cefepime
4	F	C	49	OD	MICU	25	3	7	*Staphylococcus aureus*	Cefepime, Vancomycin
5	F	AA	76	AAA	SICU	23	4	9	*Stenotrophomonas maltophilia*	Trimethoprim-sulfamethoxazole
6	F	C	20	MVC	SICU	26	6	8	*Staphylococcus aureus*	Cefepime, Vancomycin
7	F	C	68	CABG	CTICU	24	19	7	*Stenotrophomonas maltophilia* and *Pseudomonas aeruginosa*	Cefepime
8	F	AA	44	PVD	SICU	37	5	6	*Acinetobacter calcoaceticus-baumanii*	Imipenem-Cilastatin
9	M	C	62	AAA	SICU	21	1	5*	*Enterobacter cloacae*	Cefepime, Vancomycin
10	F	C	47	MVC	SICU	22	6	8	*Escerischia coli, Haemophilus influenzae* and *Streptococcus pneumoniae*	Acyclovir, Vancomycin
11	F	AA	33	GSW	SICU	17	3	7	*Haemophilus influenzae*	Cefazolin, Piperacillin-Tazobactam
12	M	C	27	MVC	SICU	16	14	5*	*Staphylococcus auerus*	Clindamycin
Validation Set										
13	M	C	29	SAH	NICU	14	n.a.	6	*Haemophilus influenzae, acinetobacter calcoaceticus-baumanii,* and *Stenotrophomonas maltophilia*	Vancomycin, Ciprofloxacin, Trimethoprim-sulfamethoxazole
14	F	C	80	CABG	CTICU	21	15	6	*Klebsiella pneumoniae*	Vancomycin, Cefepime, Metronidazole, Fluconazole
15	F	C	64	stroke	NICU	21	n.a.	6	*Streptococcus pneumoniae#*	Penicillin then Vancomycin
16	F	C	30	MVC	SICU	18	16	7	*Staphylococcus aureus*	Vancomycin, Cefepime
17	M	C	59	SAH/SDH	NICU	29	n.a.	5	*Staphylococcus aureus*	n.a.
18	F	C	50	MVC	SICU	20	"possible"	7	*Staphylococcus aureus*	Vancomycin, Ciprofloxacin, Metronidazole, Ceftriaxone
19	F	C	25	MVC	SICU	21	"possible"	9	*Streptococcus anginosus*	Cefazolin

M = Male, F = Female

C = Caucasian, AA = African-American

COPD = Chronic obstructive pulmonary disease, MVC = motor vehicle crash (trauma), CHI = closed head injury, OD = acetaminophen overdose, AAA = abdominal aortic aneurysm, CABG = coronary artery bypass grafting, PVD = peripheral vascular disease, GSW = gun shot wound, CVA = cerebrovascular accident, CABG = coronary bypass grafting, SAH = subarachnoid hemorrhage, SDH = subdural hemorrhage

culture results are from airway specimens obtained invasively (tracheal secretions or bronchoalveolar lavage)

# indicates bacterium intermediate sensitivity or insensitive to initial antibiotic treatment

CPIS = clinical pulmonary infection scores (* indicates missing P/F ratios)

n.a. = not applicable

**Figure 2:**
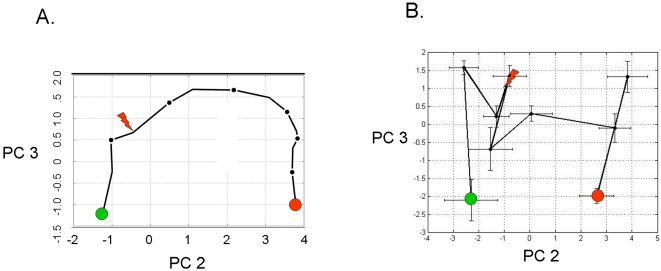
(A) Principal components analysis of the leukocyte average relative RNA abundance of the 109 human orthologs to the 219 murine genes identified in Supplemental Table S1, plotted for all eleven patients who developed VAP. The translation along principal component (PC) 2 appears to be associated with the development and recovery from pneumonia. The red arrow indicates the day where the attending physician diagnosed VAP. The green circle indicates the point at which the patient entered the study; the red circle is the point at which the patient exited the study. (B) Principal components analysis of the average absolute abundance of plasma cytokines and soluble receptors during the study period across all eleven VAP patients. Individual cytokines do not have significant changes in abundance during the time course of disease (P > 0.05 for all individual proteins).

RNA isolated from patient samples was of high quality and hybridizations met standard performance criteria (*vide supra*). To assess whether the genes identified in the murine model conveyed information in the patient study, the microarray abundance of the human orthologs of the 219 genes that distinguished the murine pneumonias were numerically analyzed. Principal components analysis of the average RMA-normalized expression levels of these 109 ortholog genes resulted in gene expression trajectories that described the cohort of patients as they developed, were treated for, and recovered from VAP (Figure 2A). Trajectory translocation along the X-coordinate (principal component 2) appeared to be informative with regard to the onset of VAP – beginning immediately before the diagnosis and ending approximately six days after appropriate antibiotic therapy was initiated. Principal components analysis of plasma cytokine abundance in these patients showed a qualitatively similar trajectory, but with large error bars (Figure 2B). Nevertheless, translocation along the X-coordinate (principal component 2) again appeared to coincide with the onset of VAP.

**Figure 3:**
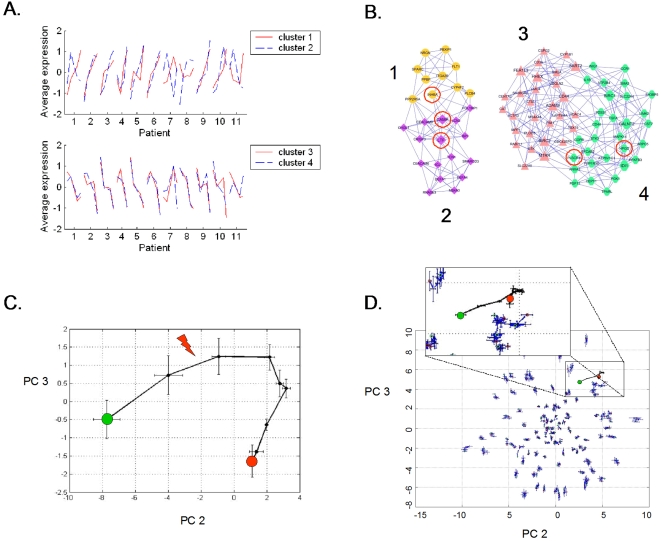
Analysis of 85 genes identified as significantly changing over time during the onset of VAP. (A) The time-dependent behavior of these genes was classified into four clusters. Shown is the normalized abundance of the average cluster member for each of the five microarrays that bracket the attending physician's diagnosis of VAP for each patient. (B) Co-expression network analysis of these four clusters was used to generate the gene expression cartography of the human blood response to acute infection superimposed on critical illness. Clusters 1 and 2 are tightly associated with one another, as are clusters 3 and 4. The red circles identify the 5 genes in common with the mouse coexpression network (Figure 1C). (C) Principal components analysis of the abundance of these 85 genes in the training data set (11 patients with VAP). PC1 (not shown) represents a constant bias term, PC2 and PC3 are shown. The arrow indicates where the attending physician's diagnosed VAP. The green and red circles indicate the points where the patients entered and exited the study, respectively. (D) Principal components analysis of microarray data generated by 100 iterations of randomly chosen sets of 85 genes, all plotted on the same two axes. The randomly chosen sets are not informational for healing from critical illness complicated by VAP. The only set that describes a discernable path is the list of 85 genes derived from EDGE analysis (inset magnified view, same riboleukogram data as in panel C).

Independent analysis of patient microarray data resulted in the identification of 85 probe sets whose abundance changed significantly during the course of VAP (Table 2). Of the 109 human orthologs that were used to calculate the trajectories shown in Figure 2A, 5 probe sets (4.6%) were present in the list of human probe sets (lactotransferrin, cathelicidin antimicrobial peptide, phospholipid scramblase 1, inhibin beta A, and hydroxyprostaglandin dehydrogenase 15-(NAD)). Network analysis found that the expression behavior of these 85 genes segregated into four clusters (Figure 3A). Transcript abundance in clusters 1 and 2 generally increased and transcript abundance in clusters 3 and 4 generally decreased around the time of VAP diagnosis.

**Table 2:** Leukocyte Genes Altered by Ventilator-associated Pneumonia.

Probe Set ID	Gene Symbol	P-Value	Q-Value	Fold Change*
202018_s_at	LOC728320///LTF	2.27E-06	0.004432733	1.464144796
207269_at	DEFA4	2.27E-06	0.004432733	1.518640915
210244_at	CAMP	1.14E-06	0.004432733	1.409490494
212531_at	LCN2	2.27E-06	0.004432733	1.427762858
206676_at	CEACAM8	4.55E-06	0.005910311	1.504273574
212063_at	CD44	4.55E-06	0.005910311	0.936827878
218095_s_at	TMEM165	6.82E-06	0.007598972	0.929587436
205033_s_at	DEFA1///DEFA3///LOC653600///LOC728358	1.48E-05	0.012805674	1.181241234
205504_at	BTK	1.48E-05	0.012805674	0.935491658
204860_s_at	LOC728519///NAIP	2.62E-05	0.018536885	0.848248535
210254_at	MS4A3	2.62E-05	0.018536885	1.441322498
204689_at	HHEX	3.41E-05	0.022163667	0.923345636
205016_at	TGFA	3.75E-05	0.022504646	0.894038802
202076_at	BIRC2	4.44E-05	0.024696657	0.968353651
207384_at	PGLYRP1	5.00E-05	0.026005369	1.152667764
207802_at	CRISP3	5.35E-05	0.026042309	1.603981321
210452_x_at	CYP4F2	6.03E-05	0.027639396	1.099086675
200665_s_at	SPARC	8.19E-05	0.029820206	1.169183993
205557_at	BPI	8.19E-05	0.029820206	1.359468336
205896_at	SLC22A4	8.42E-05	0.029820206	0.903961604
209369_at	ANXA3	8.30E-05	0.029820206	0.945826874
214146_s_at	PPBP	7.39E-05	0.029820206	1.120758241
209205_s_at	LMO4	9.44E-05	0.031992771	0.932224575
219375_at	CEPT1	0.0001126	0.03657005	0.94093974
209193_at	PIM1	0.0001228	0.038298816	0.929182785
211200_s_at	EFCAB2	0.0001331	0.038417023	0.953743854
217788_s_at	GALNT2	0.0001331	0.038417023	0.851597039
219628_at	ZMAT3	0.0001387	0.038628105	0.934196425
202464_s_at	PFKFB3	0.0001478	0.039741748	0.90582982
204068_at	STK3	0.0001933	0.044327334	0.888056674
204554_at	PPP1R3D	0.0001786	0.044327334	0.936918267
210140_at	CST7	0.0001774	0.044327334	0.935788854
212136_at	ATP2B4	0.0001876	0.044327334	0.908700634
39402_at	IL1B	0.0001831	0.044327334	0.895031859
201798_s_at	FER1L3	0.0002138	0.046244191	0.838135068
206488_s_at	CD36	0.0002172	0.046244191	0.898756001
219607_s_at	MS4A4A	0.0002195	0.046244191	0.8064475
203757_s_at	CEACAM6	0.000232	0.047593558	1.342306036
200673_at	LAPTM4A	0.0002638	0.052738161	0.954754301
202530_at	MAPK14	0.0003548	0.059983371	0.956537364
204099_at	SMARCD3	0.0003435	0.059983371	1.108065397
204225_at	HDAC4	0.0003537	0.059983371	0.927059336
204620_s_at	VCAN	0.0003594	0.059983371	0.917399007
205074_at	SLC22A5	0.0003184	0.059983371	0.926045842
206565_x_at	SMA3	0.0003491	0.059983371	0.837007379
212592_at	IGJ	0.0003617	0.059983371	1.196011695
212737_at	GM2A	0.0003514	0.059983371	0.914644647
209288_s_at	CDC42EP3	0.0003719	0.060395992	0.96733788
205098_at	CCR1	0.0003878	0.06134903	0.925839976
205110_s_at	FGF13	0.0003935	0.06134903	0.832214064
201432_at	CAT	0.0004162	0.062399247	0.96794903
205513_at	TCN1	0.0004162	0.062399247	1.301185411
202087_s_at	CTSL1	0.0004322	0.062550793	0.935466979
206838_at	TBX19	0.0004333	0.062550793	0.940074355
202437_s_at	CYP1B1	0.0004515	0.063992551	0.874011817
206493_at	ITGA2B	0.0004708	0.065541129	1.140129423
202381_at	ADAM9	0.0005061	0.066195485	0.896803767
208881_x_at	IDI1	0.0005095	0.066195485	0.919817185
214177_s_at	PBXIP1	0.0005004	0.066195485	1.061731802
222033_s_at	FLT1	0.0004947	0.066195485	1.03738168
218854_at	DSE	0.0005345	0.068307695	0.905149756
210511_s_at	INHBA	0.0005493	0.069064846	1.103622639
202187_s_at	PPP2R5A	0.0005629	0.069657239	1.041391358
201312_s_at	SH3BGRL	0.0006232	0.073610239	0.939968882
219358_s_at	CENTA2	0.0006073	0.073610239	0.911233169
220865_s_at	PDSS1	0.0006175	0.073610239	0.88394257
218699_at	RAB7L1	0.0006403	0.074496385	0.880551181
209396_s_at	CHI3L1	0.0006619	0.075877966	1.23586215
204081_at	NRGN	0.0007256	0.081973446	1.109969097
202185_at	PLOD3	0.0007642	0.085108481	0.94677975
200738_s_at	PGK1	0.0008063	0.085779381	0.977196602
202193_at	LIMK2	0.0007927	0.085779381	0.920601355
202872_at	ATP6V1C1	0.000795	0.085779381	0.900746363
202974_at	MPP1	0.0008143	0.085779381	0.963344703
202446_s_at	PLSCR1	0.0008348	0.085855047	0.953849782
204490_s_at	CD44	0.000837	0.085855047	0.88577208
203895_at	PLCB4	0.000953	0.093530674	1.031696261
206851_at	RNASE3	0.0009599	0.093530674	1.189718607
211963_s_at	ARPC5	0.0009246	0.093530674	0.980350825
215884_s_at	UBQLN2	0.0009428	0.093530674	0.906980156
201810_s_at	SH3BP5	0.0010099	0.097191784	0.921374384
211548_s_at	HPGD	0.0010224	0.097195788	0.755385963
200996_at	ACTR3	0.001077	0.099814726	0.97523089
201358_s_at	COPB1	0.0010861	0.099814726	0.96732026
203200_s_at	MTRR	0.0010884	0.099814726	0.864551148

* Change from day 3 to day −3, relative to the VAP diagnosis at day 0

Molecular cartography of the human leukocyte transcriptional response to acute bacterial infection identified two densely connected networks of genes, the first containing clusters 1 and 2 and the second containing clusters 3 and 4 (Figure 3B). The 26 probe sets in clusters 1 and 2 are significantly enriched with GO biological process terms: “defense response to bacteria” (8 genes, p-value = 2×10^−11^), “response to biotic stimulus” (12 genes, p = 4×10^−6^), and “immune response” (8 genes, p = 0.002) and the cellular compartment term “extracellular region” (14 genes, p = 9×10^−9^). The 59 probe sets in clusters 3 and 4 are enriched with GO molecular function terms: “ATP binding” (13 genes, p = 9×10^−4^), “metal ion binding” (22 genes, p = 0.002), and “protein binding” (25 genes, p = 0.002) and the cellular compartment terms “cytoplasm” (23 genes, p = 0.007) and “plasma membrane” (13 genes, p = 0.02).

Principal components analysis of the microarray expression profiles of these 85 genes defined a common response to pulmonary infection complicating critical illness (Figure 3C). Importantly, trajectory translocation in the X-coordinate (principal component 2) occurred days prior to the clinical diagnosis of VAP. In addition, the informational content of 85 genes chosen at random was determined iteratively 100 times for the first 11 VAP patients. As shown in Figure 3D, only the 85 genes identified by EDGE as significant (FDR≤0.10) produced a trajectory; the other sets of genes were scattered randomly around the origin of the graph.

**Figure 4:**
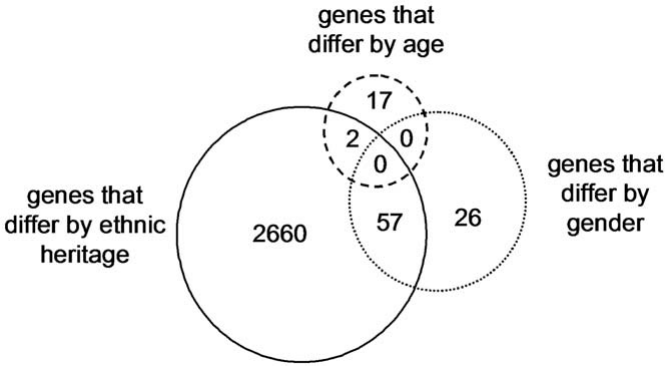
Genes informational in distinguishing clinical phenotypes of interest, including host gender, age, and ethnic background, with the caveats that all African-Americans and all middle-aged individuals were female (Table 1). Note that the largest number of probe sets were associated with differences in ethnic background (African-American compared to Caucasian). Of note, as opposed to the mouse response, there we no genes that differentiated between the human response to bacterial cell wall products (Gram negative versus Gram-positive bacteria), suggesting that signal variance due to ethnic background, gender, and age is greater than that due to infecting organism.

Three patients exhibited contrary gene expression profiles within two of these four clusters (Figure 3A). Patients 1, 7 and 11 showed decreased expression of genes in cluster 2 and patients 7 and 11 showed increased expression of genes in clusters 3 and 4. Based on patient demographics (Table 1 and data not shown), the only clear difference between patients 7 and 11 and the other patients in the study is that these two patients developed VAP later in their ICU course (study day 18 and 14 respectively) whereas the remaining patients developed VAP between study days 3 and 6. This was also evident in PCA analysis of the 11 individual trajectories (data not shown). The changes in transcript abundance of selected genes were validated by quantitative RT-PCR (Figure S1). Finally, we tested whether host ethnicity, host gender, host age, or the cell wall phenotype of the infectious agent had an effect on the number of informational genes. The most informational of these demographic variables was host ethnicity (Figure 4).

**Figure 5:**
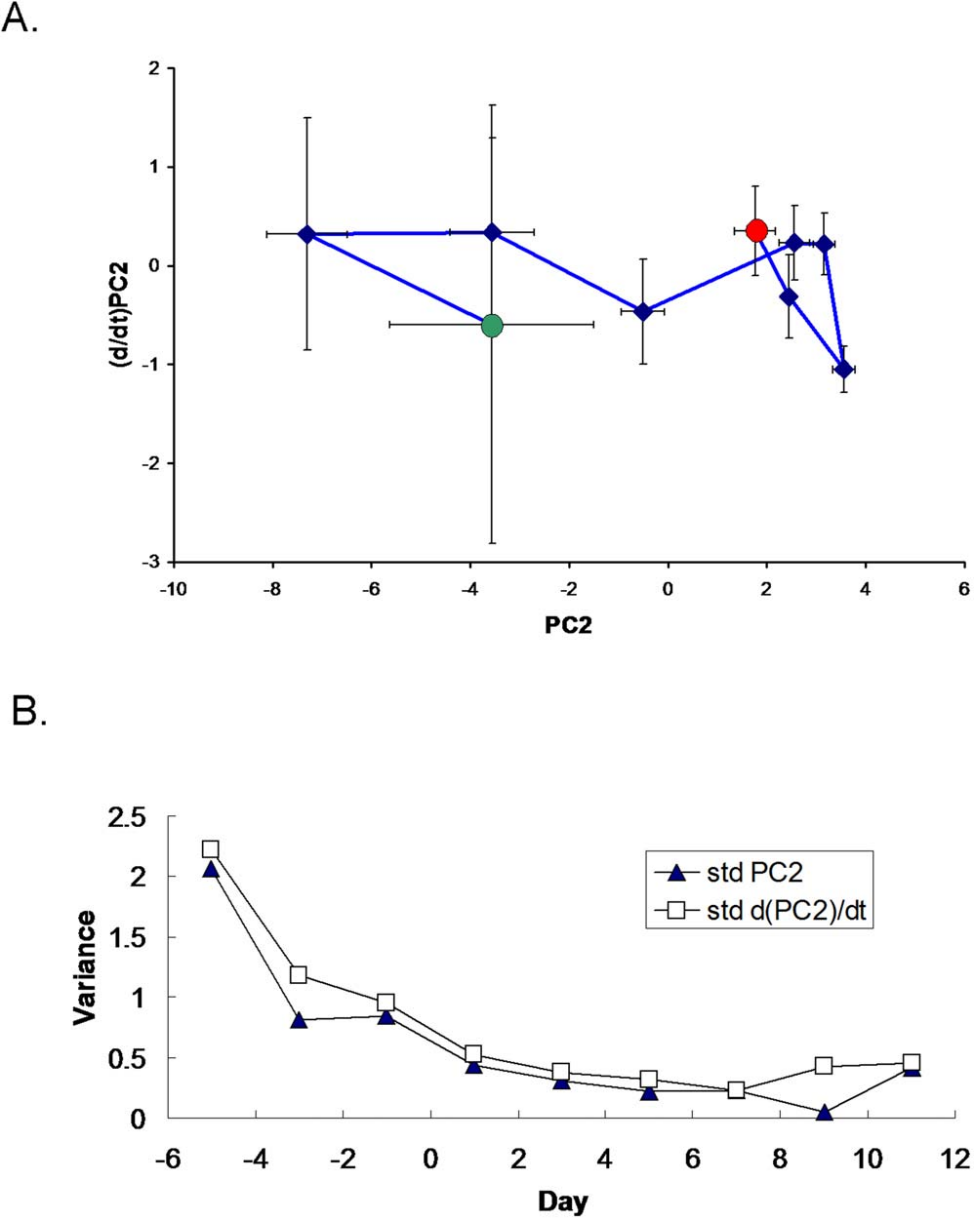
Phase space analysis of the average ICU patient riboleukogram trajectories as they develop VAP, respond to antibiotics, and recover. (A) Decrease of variance and the convergence of individual trajectories to a common small region in the phase space (“immunological attractor”) associated with health. The green and red circles indicate where the patients entered and exited the study, respectively. (B) Decreases in variance (standard deviation, STD) over time for the phase space trajectory in panel A, consistent with the existence of an attractor. The diagnosis of VAP was made by the attending physician on Day 0.

We observed in Figure 3C that the aggregate riboleukogram variance in principal component 2 decreased as patients recovered from acute infection. This finding suggested that principal components analysis of microarray-measured gene expression described an attractor, as gene expression time series can be described in terms of dynamical system theory as trajectories in the phase space defined by the main principal components. By plotting the change in PC2 against PC2 over time, we found indeed that the mapped gene expression information appeared to converge toward a common point, suggesting that PC2 represents the expression of the infection-inducible genes (Figure 5). Consistent with differences in patient age, gender, ethnicity, pre-existing co-morbidities, and nature of injury insult, each patient's individual trajectory started at a different point and described a patient-specific arc (data not shown).

### Validation cohort

**Figure 6:**
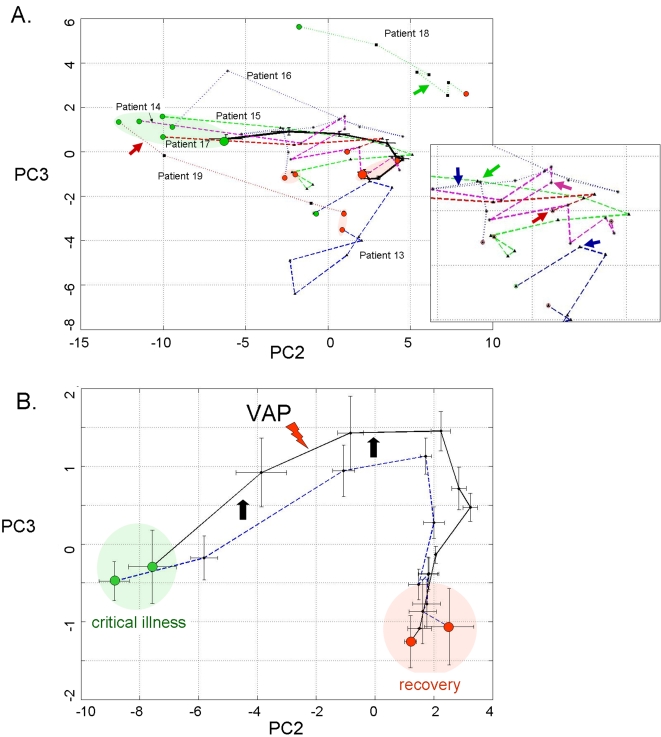
Principal components analysis of 85 leukocyte genes in the training and validation patient cohorts. (A) The solid black curve depicts the aggregate riboleukogram of the first 11 VAP patients (training cohort, same data as in Figure 3C). The other 7 curves are the individual riboleukograms of the patients in the validation cohort. The inset magnifies the trajectories of patients 13–17 (see Table 1) and demonstrates abrupt changes in riboleukogram course typically coincident with an increase in CPIS score (first occurrence of maximal CPIS value is indicated by the arrows). The paths of patients 13, 18 and 19, are atypical (see text for additional details). (B) The aggregate 11 patient VAP riboleukogram (black curve, same as panel A) is compared to the aggregate riboleukogram of all patients aligned by study day (that is, training and validation cohorts, irrespective of VAP day of diagnosis, dotted blue curve). Note that the VAP riboleukogram deviates from the “critical illness” riboleukogram (black arrows) prior to VAP diagnosis (lighting bolt), but after treatment, the VAP riboleukogram converges with the critical illness riboleukogram at the point of recovery. The green and red circles indicate where the patients entered and exited the study, respectively.

A second cohort of 7 patients was analyzed to evaluate the informational value of the 85 genes that were identified in the first 11 patients with VAP. Two of these 7 additional patients were diagnosed with “late” VAP by the attending physician, while another 2 cases were described by the attending as “possible” VAP (Table 1). The individual riboleukograms for these 7 patients demonstrate the existence of immune recovery (basins of attraction) as well as the heterogeneity of the host response. In general, the individual riboleukograms follow a path moving from left to right, that is, from critical illness to recovery (Figure 6, green and red shaded areas, respectively). The development of an infectious complication is typically associated with a change in riboleukogram trajectory. For example, the paths of patients 13, 14, 15, 16, and 17 change directions abruptly coincident with changes in clinical status and concern for VAP or sepsis (see Figure 6A inset). Patient 17 grew *Staphylococcus aureus* from both urine and tracheal secretions prior to withdrawal of therapy for cure (the only death in the study). In contrast, the riboleukograms of patients 13, 18, and 19 are atypical, in that their paths do not start and/or do not finish with the others. Both patients 18 and 19 had pulmonary contusions secondary to polysystem trauma, maximal CPIS scores of 7 and 9, Gram-positive cocci cultured from airway secretions, and were treated with antibiotics, but had a clinical course labeled by the attending physician as “possible” VAP. Their riboleukograms are in different portions of the graph in Figure 6A, but have a similar shape and slope. Both patients 13 and 18 had intracranial hemorrhage. Patient 13 was not diagnosed with VAP (no infiltrate on CXR) but was treated with antibiotics for a fever of 39.4°C and WBC of 31,300 (day 5), tracheal secretions that subsequently grew out Acinetobacter and Stenotrophomonas (CPIS 6), and concern for catheter-related sepsis.

In Figure 6B, the aggregate riboleukogram for the training cohort of VAP patients (aligned for day of VAP diagnosis, see also Figure 3C) is compared to the aggregate riboleukogram for all patients aligned for day of study entry (that is, both training and validation cohorts, N = 11+7, irrespective of VAP day). Again noted are the PCA domains of critical illness and recovery. The aggregate VAP riboleukogram diverges from the aggregate critical illness riboleukogram and rejoins the latter at the point of recovery.

## Discussion

Using a bench-to-bedside approach, we have implemented a mouse model of pneumonia and found that RNA abundance profiles obtained from blood samples taken prior to appreciable mortality were able to distinguish between the two variables tested in the assay: lethality of the insult and type of infectious agent. These data extend our previous observations in a mouse model of abdominal sepsis, wherein microarray-measured expression profiles from circulating leukocytes distinguished between infectious and non-infectious etiologies of systemic inflammation in a de-identified cohort [15]. Thus, the mouse circulating leukocyte transcriptional response to infection can not only distinguish between infectious and non-infectious inflammatory insults, but also the type of infectious agent and its associated mortality. Network analysis suggested that the pneumonia-induced transcriptional changes reprioritized mouse leukocytes for the initiation of an immune response, the transcriptional regulation of intracellular signaling cascades, and the induction of numerous transcription factors and other nuclear genes.

Results from the mouse model suggested that the transcriptional activity of buffy coat-isolated cells may be used to monitor the onset of and recovery from acute infection. We tested this hypothesis by calculating principal components using microarray expression profiles of RNA isolated from mechanically ventilated patients at risk for pneumonia. Initially, we examined the behavior of the human orthologs to the genes identified in the mouse pneumonia study. The onset of acute infection corresponded with translation along PC 2 in Figure 2. Translation along PC2 ceased 5–6 days after appropriate antibiotic therapy in the patients was started, consistent with recovery. These data show that there are specific transcriptional programs instituted by circulating immune cells during acute infection which have diagnostic potential in the setting of critical illness. Principal components analysis of plasma cytokine abundance generated a qualitatively similar trajectory; however, in line with previous reports, plasma cytokine abundance (including procalcitonin) was insufficient to diagnose acute infection in this small cohort of critically ill patients. As with other tissues, changes in RNA abundance observed in circulating leukocyte do not necessarily reflect changes in protein abundance, and vice versa. While principal components analysis of informational murine genes in authentic human disease showed there was a conserved and informational peripheral leukocyte transcriptional response to localized infection, the information contained in those genes would not appear to be more useful than current clinical criteria (that is, the translation in PC2 did not begin until the day the attending physician made the diagnosis). However, by explicitly accounting for variance over time, a set of 85 genes were identified subsequently in our first 11 VAP patients whose microarray expression levels changed consistently before the clinical diagnosis of VAP.

These 85 genes clustered into four groups with their abundance either increasing or decreasing throughout the 7-day window bracketing the onset of infection (Figure 3A). There was a significant association between genes known to play key roles in defense against bacterial pathogens and those genes that increased in apparent abundance (cluster 1 and 2 probes sets) coincident with the diagnosis of VAP. Consistent with activation of the host response to pneumonia, all of the genes with the “defense against bacteria” ontology were found in cluster 2 and encode primarily granulocytic, antimicrobial proteins, and adhesion molecules. In contrast, the genes that decreased in apparent abundance (clusters 3–4) showed different behavior depending on whether the patient developed VAP early or late in the study. Although no consistent biological theme emerged from this list of the 59 transcripts, this finding provides some insight into the transcriptional basis of differences in the critically ill host's response to early- *versus* late-onset VAP [29]. Of interest, the RNA abundance of five genes were altered by pneumonia in both the mouse and human gene sets (Figures 1C and 3B): lactotransferrin (LTF), cathelicidin antimicrobial peptide (CAMP), phospholipid scramblase 1 (PLSCR1), inhibin beta A (INHBA), and hydroxyprostaglandin dehydrogenase 15-(NAD) (HPGD). Pathway analysis indicates that these five genes connect in a network rich with interactions between important mediators of inflammation, as seen in Figure S2 and Table S2, (Ingenuity Pathway Analysis, Ingenuity Systems, Redwood, CA). Principal components analysis of the microarray expression data for the 85 transcripts generated a curve with qualitative similarities to curves derived from either cytokines or the 109 human orthologs; however, the patient-to-patient variance in the measured abundance of these genes was significantly smaller and translation along the second principal component preceded the diagnosis of VAP by 24 to 72 hours (Figure 3C). In contrast, equivalent analysis of randomly selected sets of genes provided no information about the host response to critical illness complicated by pneumonia.

Macroscopically, pneumonia is diagnosed when there are symptomatic changes in clinical status, as manifested typically by increased CPIS scores. However, microscopically pneumonia occurs at the transition from colonization to infection concomitant with failure of multiple host barriers protecting the bronchoalveolar epithelium. These events lead to tissue exposure to bacteria and bacterial products, and in many cases to toxicosis and bacteremia. Our data suggest that during VAP, circulating leukocytes are exposed to bacterial products 24–72 hours prior to diagnosis by an attending physician. Initiation of antibiotic therapy earlier during this time window is expected to significantly improve outcome [16]. These findings were further evaluated in a small validation patient cohort, confirming the value of the 85-gene riboleukogram to quantitate the host response to and recovery from critical illness. Individual riboleukograms changed direction coincident with clinical findings of pneumonia or sepsis (frequently days before the clinical diagnosis and maximal CPIS scores). Nevertheless, marked heterogeneity was observed in some patient responses that could not be linked to monitored variables (*e.g.*, patients 13 and 18, Figure 6A). In these patients, perhaps the well-described influence of the underlying acute illnesses (pulmonary contusion and intracranial hemorrhage) on host immune responses could provide part of the explanation [30], [31]. As patients healed from critical illness, the riboleukograms converged, a finding consistent with the existence of an immunological “attractor” state. Comparison of the aggregate VAP *versus* critical illness riboleukograms indicated that a large portion of the signal for the 85 genes identified is a reflection of recovery from critical illness (PC2 in Figure 6B). Nevertheless, VAP signal became evident among the “noise” of critical illness once the individual riboleukograms were aligned for day of VAP diagnosis, evident as a deflection of the VAP riboleukogram upward in PC3 (Figure 6B). Thus, we submit that riboleukograms are a molecular analytical tool with substantial potential to improve diagnostics, prognostics, and our understanding of the host response to critical illness complicated by acute infection.

An important consequence of our observation that patient-specific riboleukograms converged is that the variance in leukocyte gene expression for these 85 genes decreased significantly over the time course in patients with VAP. Studies of physical systems suggest that the stable states of networks can be represented as attractors, a set of points in the phase space to which the genetic network evolves over time [32], [33]. In particular, every trajectory initiated within the bounds of an attractor terminates inside the attractor. Recently this hypothesis has been confirmed experimentally *in vitro* [11], [34]. Our findings suggest that the immune system of a critically ill patient who recovers from disease returns back to a stable state and that the immune response trajectory can be a considered formally as a dynamic system. Borrowing from concepts in the physical sciences [35], we hypothesized that an individual's baseline immune system is within a basin of attraction prior to injury, nosocomial infection perturbs that system, and after the infection cleared the system would return to it's initial attractor. In phase space analysis, we found that the patient-specific trajectories appeared to converge and that the onset of pneumonia ‘pushes' the gene expression data away from that basin—a perturbation that is ameliorated by appropriate antibiotic therapy. This result provides what we believe is the first evidence in patients for a basin of attraction for the genetic network associated with immunological health.

Because of the heterogeneity of this patient population (for example, age, gender, pre-existing health status, type and severity of critical illness), it is not surprising that the error bars on the PC analysis are initially quite large. The end-points of the trajectories, however, appear to converge in a smaller space, suggesting that the patients' immune systems are returning to a health attractor (Figures 5 and 6). As observed in the validation cohort, patient-specific trajectories are not smooth curves, but appear to have inflection points that may correlate with hospital intervention or the onset of infection. Our data further suggest that differences in host ethnic backgrounds and gender are more important than differences in infecting organism as determinants of the host leukocyte transcriptional response. These observations are consistent with a recent study comparing GeneChip signal from cells lines derived from Asians and Caucasians, indicating differences in the gene expression levels of 25% of the 4000 genes studied in these two ethnic groups [36]. Moreover, as the rate of sepsis, the youngest age of sepsis onset, and the sepsis mortality rate are highest for African-American males [37], our data suggest that further study of riboleukograms is indicated to gain insight into these health disparities. These profiles also suggest that there is a transcriptional component that is conserved across that diversity of the human population.

In summary, our analysis demonstrates the plasticity of the blood leukocyte response to bacteria *in vivo,* extending previous *in vitro* studies [10], [11] into the clinical realm. For the first time, we provide evidence that riboleukogram gene expression analysis can be applied to a heterogeneous clinical population to monitor the host response to and recovery from critical illness complicated by acute infection. Moreover, we identify new, conserved gene targets that appear to be informational for recovery at the transcriptional level, many of which are involved with granulocyte maturation and chemokine (not cytokine) responsiveness. This conclusion is supported by the relative weak diagnostic information provided by the plasma cytokine data, consistent with conclusions reached at recent a consensus conference [16]. In particular, plasma procalcitonin levels were not informational. There are, however, important limitations to our study, most notably, the preliminary nature of the work, the small number of patients, and the well-known difficulty of ruling out false-positive and false-negative diagnoses of VAP based on clinical parameters. This uncertainty is both the motivation for (and challenge of) developing novel molecular diagnostics for VAP. In addition, there is no consensus on how to leverage the dynamic nature of the clinical, RNA, and protein data collected to build hybrid models that improve diagnostics and prognostics. Thus, we conclude that our data demonstrate the technical feasibility and clinical potential of the riboleukogram approach, but proof of clinical utility will require further study.

Thus, as graphs of myocardial electrical information (electrocardiograms) were tapped over a century ago to provide an objective means to aid heart diagnostics, we submit that riboleukograms will aid in the diagnosis and prognosis of acute infectious and inflammatory disease. The diagnostic potential of riboleukograms is supported by two very recent independent reports that corroborate our mouse data (Figure 1D), indicating that circulating leukocyte RNA signatures in patients differentiate between the host responses to sterile *versus* infectious causes of systemic inflammation and between the host response to Gram-negative *versus* Gram-positive pathogens [38], [39]. Prospective clinical trials are indicated to validate our results and determine the value of this new technology; to optimize gene selection methods that account for differences in patient ethnicity, gender, and age; and to develop computational approaches that integrate clinical and molecular data to improve diagnostics [40].

## Supporting Information

Figure S1 The changes in transcript abundance of selected genes from Table 2 were validated by quantitative RT-PCR. For the six genes depicted above, the RT-PCR correlation coefficients (R2 values) for the microarray expression values are 0.60, 0.67, 0.66, 0.86, 0.85, and 0.43, respectively.
(0.32 MB TIF)

Figure S2 Hypothetical leukocyte network based on reported gene-gene interactions. Starting with the 5 genes altered by pneumonia in both mice and patients (grey shapes), the Ingenuity Pathway Analysis tool was used to build an interaction network based upon gene-gene interactions reported in the literature. Note the rich network of interactions and the consistent inflammation theme of the genes added by the software. Table S2 lists the individual genes and their reported connections.
(3.24 MB TIF)

Table S1 219 mouse genes: probe/gene mean fold change
(0.08 MB XLS)

Table S2 Rich network connections for 5 common mouse/human genes (Ingenuity Pathway Analysis)
(0.02 MB XLS)
